# What drives food choices? Sociodemographic predictors in a representative Czech population sample

**DOI:** 10.1186/s12889-026-26926-z

**Published:** 2026-03-09

**Authors:** Monika Kunzová, Leona Mužíková

**Affiliations:** 1 Department of Public Health, Faculty of Medicine, Masaryk University, Brno, Czech Republic; 2 International Clinical Research Center of St. Anne’s University Hospital Brno, Brno, Czech Republic; 3 Department of Physical Education and Health Education, Faculty of Education, Masaryk University, Brno, Czech Republic

**Keywords:** Food choices, Socioeconomic factors, Health behavior, Czechia, Central and Eastern Europe

## Abstract

**Background:**

Dietary behavior represents a major determinant of population health, contributing substantially to obesity, diabetes, cardiovascular disease, and other non-communicable conditions. Across Central and Eastern Europe, dietary risks are compounded by socioeconomic inequalities and post-socialist market transitions that have reshaped food environments. While price, taste, and health are well-established drivers of food choice, less is known about how these motives, including emerging environmental considerations, are distributed across social groups in this region.

**Aim:**

This study examined how sociodemographic characteristics are associated with food choice motives among Czech adults in a nationally representative sample, and how these patterns relate to broader regional contexts.

**Methods:**

A cross-sectional survey of 1,803 Czech adults (aged ≥ 15 years) was conducted using quota sampling representative by age, gender, education, region, and settlement size. Respondents selected up to three key food choice criteria from a standardized list. Associations between gender, age, education, and income and each motive were assessed using multivariable logistic regression.

**Results:**

Taste (63.7%), price (37.6%), and health considerations (31.5%) were the most frequently reported food choice motives, whereas environmental considerations were rare (2.5%). Men more frequently reported taste-based motives, while women more often emphasized health- and nutrition-related considerations. Higher education was associated with greater attention to nutritional value and ingredient quality, whereas lower education and income were linked to greater price sensitivity and reliance on hedonic cues. Environmental considerations showed no strong sociodemographic patterning.

**Conclusion:**

Food choice motives in post-socialist Central Europe are shaped by multiple sociodemographic factors, with persistent socioeconomic gradients. Addressing these disparities may require policies that prioritize structural and economic determinants of food choice while cautiously integrating environmental objectives, in order to align equity, public health, and sustainability goals.

**Supplementary Information:**

The online version contains supplementary material available at 10.1186/s12889-026-26926-z.

## Introduction

Dietary behavior is a primary determinant of population health, accounting for nearly one in five global deaths through its contribution to obesity, diabetes, cardiovascular disease, and other non-communicable conditions [1, 2]. In Europe, suboptimal diets remain the leading modifiable risk factor for morbidity and premature mortality, despite decades of health promotion efforts [3]. The structure of modern food environments—dominated by ultra-processed, energy-dense, and aggressively marketed products—has normalized patterns of overconsumption and nutritional imbalance [4]. 

Across Central and Eastern Europe, these challenges have been further shaped by widening socioeconomic inequalities and profound market transformations following the political and economic transitions of the 1990s [5]. In many Central and Eastern European countries, dietary patterns have shifted away from traditional foods toward industrially processed, price-competitive alternatives, a transition that has accompanied steep rises in obesity and metabolic disease [5, 6]. Although dietary risk factors are well documented, less attention has been paid to the motivational and sociocultural underpinnings of these behaviors—how individuals, shaped by gender, age, education, and income, navigate increasingly complex food environments.

Research on food choice motives has predominantly focused on Western European or high-income contexts, where cultural norms, market maturity, and public health infrastructure differ substantially from post-socialist societies [7–9]. Consequently, the behavioral and structural mechanisms underlying dietary decisions in Central and Eastern Europe remain insufficiently examined. Although cross-national studies have identified socioeconomic gradients in food habits across Europe, they rarely capture the complex interplay of social stratification, cultural heritage, and historical legacies that continue to shape dietary patterns in this region [5]. 

In Czechia, nationally representative evidence on food choice motives is virtually absent. Previous research has concentrated primarily on nutritional intake, food insecurity, or dietary quality, but not on food decision-making’s motivational and psychosocial dimensions [10, 11]. Given the country’s position as one of the most industrialized post-socialist economies and its ongoing dietary transition, Czech data provide a critical case study for understanding the broader Central European context.

Although price, taste and health are well-established global determinants of food choice, nationally representative studies examining how these motives distribute across demographic strata are rare in Central and Eastern Europe [12, 13]. Similarly, although environmental and sustainability considerations are now central to European nutrition policy, their expression in population-level analyses remains limited in Central and Eastern Europe [14]. A deeper understanding of these motivational gradients is crucial for designing equitable and culturally resonant nutrition policies aligned with the European Green Deal and the Farm-to-Fork Strategy objectives [15, 16]. 

While sustainability has become a central concept in European food and nutrition policy, it is important to distinguish between its multidimensional theoretical definition and the specific motivational constructs that can be empirically captured in population surveys. Sustainability in food systems typically encompasses environmental, social, and economic dimensions, including environmental impact, social justice, labour conditions, and affordability [14–16]. 

The present study does not aim to measure sustainability in its full conceptual breadth. Instead, it focuses on environmental considerations in food choice, understood as individual attention to the environmental impact of food products. This construct represents one frequently invoked component of sustainability discourse, but it should not be interpreted as a comprehensive indicator of sustainable food consumption [14, 17, 18]. 

Conceptually, this study draws on models of food choice that emphasize the interaction between individual preferences and broader structural constraints. The food choice process framework and life-course perspectives conceptualize food choice as a dynamic outcome of personal values, socioeconomic resources, and the surrounding food environment, rather than as a purely rational or value-driven decision [7, 12, 19]. Within this framework, environmental considerations are treated as one possible, but not dominant, motivational input competing with more immediate drivers such as price, taste, and health. These perspectives align with previous research conceptualizing food choice as the outcome of interacting personal motives, social context, and structural constraints, rather than a purely value-driven or rational process [20]. 

Against this background, the present study analyzed how sociodemographic factors shape the motivational landscape of food choice among Czech adults, using data from a nationally representative sample. Specifically, it sought to identify dominant food choice motives across gender, age, education, and income groups; assess the presence and sociodemographic distribution of environmental considerations in dietary decision-making; and interpret these findings within the broader sociocultural and policy context of Central and Eastern Europe. The present study examines how sociodemographic characteristics are associated with food choice motives among Czech adults in a nationally representative sample and offers insights for developing public health strategies by considering different target audiences based on sex, age, and other sociodemographic and contextual variables.

## Methods

### Design and population

This cross-sectional study was conducted in the Czech Republic between November 20 and December 6, 2023. The research formed part of a broader sociological survey on food-related behaviors and consumer preferences. The study population consisted of residents of the Czech Republic aged 15 years and older. A total of 1,812 individuals were initially interviewed, and 1,803 respondents were included in the final analysis after excluding cases with missing data. The study design followed established sociological research standards and employed validated instruments developed by the research team.

### Sampling and recruitment

Participants were selected using quota sampling, designed to align the sample with the Czech population in terms of age, sex, education, region, and residence size. The quotas were derived from national demographic statistics of the Czech Statistical Office. Data were collected by 207 trained professional interviewers from the INRES Agency, who underwent standardized instruction prior to fieldwork. All participants provided informed consent before participation, and their responses were collected anonymously. While quota sampling ensured alignment with population margins for age, sex, education, region, and settlement size, it does not fully eliminate the potential for selection bias and does not guarantee unbiased population representativeness.

### Data collection

A structured interviewer-administered questionnaire was developed by the research team specifically for this sociological survey. The instrument covered sociodemographic characteristics, food-related behaviours and the key motives for food choice. Its content was informed by previous literature on food choice motives and dietary behaviour, including established frameworks assessing food choice motivations and consumer attitudes toward health and sustainability [7, 14, 21]. Prior to the main survey, the questionnaire was pilot-tested in a sample of 206 respondents to assess clarity, comprehensibility and cultural appropriateness; minor wording adjustments were made based on the pilot results. The full English version of the questionnaire and detailed results of the pilot testing are provided in Supplementary Material 1. Data were gathered electronically using secure data entry software and subsequently cleaned and validated by the research team before analysis.

### Variables definition

The primary outcome variables were food choice criteria, including appearance, taste, nutritional value, health considerations, price/affordability, quality of ingredients, and environmental considerations. The food choice criteria were operationalized as self-reported motives guiding food selection. Each criterion was measured using a single questionnaire item, and respondents were asked to select up to three criteria they considered most important when choosing food.

Environmental considerations were captured by a single item referring to attention to the environmental impact of food products. This item was intended to reflect individual environmental considerations in food choice rather than sustainability as a broader multidimensional construct.

Sociodemographic variables included gender, age, education, and household income. Region of residence was recorded for descriptive purposes. Education was categorized as primary, upper-secondary vocational, upper-secondary general, post-secondary, and tertiary. Monthly household income was reported in euros per month and grouped into three categories: <€1200, €1200–2500, and >€2500. Income was reported as total monthly household income and was not equivalized for household size.

### Ethics approval and consent to participate

The study was conducted in accordance with the 1964 Helsinki Declaration and its later amendments. Participation was voluntary, and respondents were informed about the purpose and anonymity of the research prior to data collection. Written informed consent was obtained from all participants. The research protocol was reviewed at the Faculty of Education, Masaryk University, and it was determined that formal ethics committee approval was not required for this type of anonymous, non-interventional sociological survey conducted in accordance with Czech national legislation (Act No. 110/2019 Coll., implementing the GDPR). The dataset was provided in fully anonymized form by the INRES agency, which was responsible for fieldwork implementation and data protection.

### Statistical analysis

All statistical analyses were performed using IBM SPSS Statistics, version 28.0 (IBM Corp., Armonk, NY, USA). Continuous variables are expressed as means and standard deviations (SD), and categorical variables as percentages with corresponding 95% confidence intervals (95% CI). Differences between proportions were examined using the chi-square test. To assess the association between sociodemographic characteristics and each food choice criterion, a series of binary logistic regression models were constructed. Each food choice variable was coded dichotomously (1 = selected, 0 = not selected). Independent variables included gender, age group, educational attainment, and household income. Results are reported as odds ratios (ORs) with 95% CI, and the level of statistical significance was set at *p* < 0.05.

The analyses were conducted using standard logistic regression models and were not adjusted for survey weights or clustering. Given the quota-based sampling design, the analyses were intended to examine associations between sociodemographic characteristics and food choice motives rather than to provide fully weighted population-level estimates or causal inference.

## Results

### Participants’ characteristics

Of the 1812 respondents recruited through quota sampling, 1803 were included in the final analysis after excluding nine with incomplete data. The sample comprised 48.5% men and 51.5% women, with a mean age of 47.8 (± 18.2) years. Participants were geographically distributed across Prague (12.5%), Bohemia (49.0%), and Moravia & Silesia (38.5%).

Overall, 6.7% had primary education, 28.3% upper-secondary vocational, 44.0% upper-secondary general or post-secondary, and 21.1% tertiary education. Household income was below €1200 in 23.2% of participants, between €1200–2500 in 51.4%, and above €2500 in 25.4% (Table 1).

**Table 1:** Participant characteristics

	*n* = 1803	% (95% CI)
Gender		
Man	874	48.5 (46.2–50.8)
Woman	929	51.5 (49.2–53.8)
Age (mean, SD)		47.8 (± 18.2)
Age categories		
≤ 29 years	386	21.4 (19.5–23.3)
30–44 years	395	21.9 (20.0–23.8)
45–59 years	487	27.0 (25.0–29.0)
≥ 60 years	535	29.7 (27.6–31.8)
Region		
Prague	225	12.5 (11.0–14.1)
Bohemia	883	49.0 (46.7–51.3)
Moravia & Silesia	695	38.5 (36.3–40.8)
Education		
Primary	121	6.7 (5.6–8.0)
Upper secondary – vocational	510	28.3 (26.3–30.4)
Upper secondary – general / post-secondary	739	44.0 (41.7–46.3)
Tertiary	379	21.1 (19.3–23.0)
Income		
< 1200 €	418	23.2 (21.3–25.2)
1200–2500 €	927	51.4 (49.1–53.7)
> 2500 €	458	25.4 (23.4–27.5)

### Food choices criteria

**Fig. 1:**
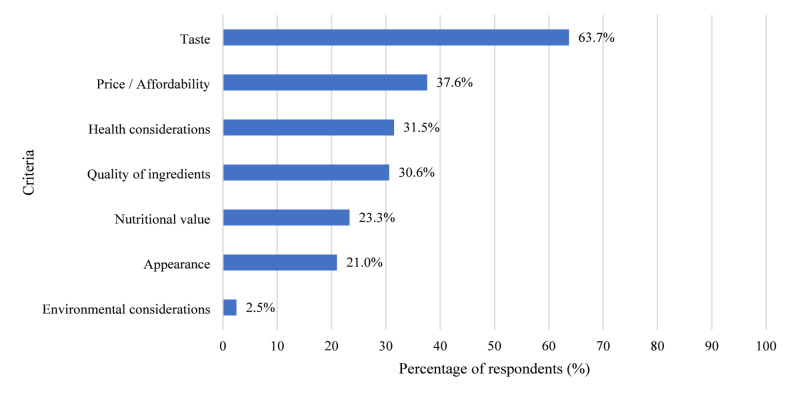
Food choice criteria among respondents

Among the assessed food choice criteria, taste emerged as the most dominant factor, reported by nearly two-thirds of respondents (63.7%; 95% CI: 61.5–65.9). Other frequently cited determinants included price and affordability (37.6%; 95% CI: 35.4–39.9), health considerations (31.5%; 95% CI: 29.4–33.7), and quality of ingredients (30.6%; 95% CI: 28.5–32.8). Nutritional value (23.3%; 95% CI: 21.4–25.3) and appearance (21.0%; 95% CI: 19.2–23.0) were mentioned less often, with environmental considerations noted by only a small percentage of respondents (2.5%; 95% CI: 1.9–3.3) (Fig. 1).

Compared with women, men more frequently reported taste as an important food choice criterion (*p* = 0.005). Women more often reported nutritional value, health considerations, price/affordability, and quality of ingredients (all *p* < 0.05). No gender differences were observed for appearance or environmental considerations (Table S1, Supplementary Material 2).

Across age groups, no substantial differences were observed for appearance or taste. Other food choice criteria showed no clear monotonic trends with age, although a modest, non-significant increase was observed for quality of ingredients. Environmental considerations remained infrequently reported across all age groups (Table S2, Supplementary Material 2).

Participants with lower educational attainment more frequently reported taste, appearance, and price/affordability, whereas those with higher education more often selected nutritional value and quality of ingredients (*p* < 0.001). A modest increase in environmental considerations was observed across educational levels, although their overall prevalence remained low (Table S3, Supplementary Material 2).

Participants with lower household income more frequently prioritised price/affordability and health considerations, whereas those with higher income more often reported nutritional value and quality of ingredients (*p* < 0.001). Environmental considerations did not differ across income categories (Table S4, Supplementary Material 2).

In multivariable logistic regression models adjusted for sociodemographic characteristics (Table 2, Fig. S1 A–G in Supplementary Material 2), women were less likely than men to report appearance-based (OR = 0.59, 95% CI: 0.44–0.79, *p* < 0.001) and taste-based (OR = 0.52, 95% CI: 0.38–0.72, *p* < 0.001) food choices. Participants with lower education were more likely to select food based on appearance and taste (*p* < 0.001), whereas higher-educated respondents more frequently reported nutritional (OR = 0.49–0.64, *p* ≤ 0.01) and ingredient-related (OR = 0.44–0.70, *p* ≤ 0.05) considerations. Price sensitivity showed strong associations with both lower education (OR = 2.93, 95% CI: 1.89–4.54, *p* < 0.001) and lower income (OR = 3.42, 95% CI: 2.21–5.31, *p* < 0.001). Environmental considerations were infrequently reported (2.5%) and were not significantly associated with any sociodemographic variable.

**Table 2:** Multivariable logistic regression models for sociodemographic determinants of food choice criteria (OR, 95% CI)

Food choice criterion	Gender		Age			Education		Income	
	Women vs. Men	30–44 vs. ≤29	45–59 vs. ≤29	≥ 60 vs. ≤29	Primary vs. Tertiary	Vocational vs. Tertiary	Upper secondary vs. Tertiary	< 1200 € vs. >2500 €	1200–2500 € vs. >2500 €
Appearance	0.59 (0.44–0.79)***	1.07 (0.71–1.61)	1.20 (0.80–1.79)	1.04 (0.68–1.61)	2.67 (1.43–4.99)**	2.69 (1.71–4.23)***	1.52 (1.05–2.19)*	1.11 (0.71–1.74)	1.26 (0.90–1.75)
Taste	0.52 (0.38–0.72)***	0.86 (0.54–1.36)	0.75 (0.48–1.17)	1.15 (0.71–1.88)	2.83 (1.31–6.11)**	4.06 (2.43–6.77)***	1.63 (1.12–2.36)*	0.66 (0.40–1.10)	0.78 (0.53–1.14)
Nutritional value	1.25 (0.94–1.66)	0.94 (0.63–1.40)	0.71 (0.48–1.06)	0.55 (0.36–0.85)**	0.49 (0.25–0.96)*	0.48 (0.31–0.75)***	0.64 (0.46–0.90)*	0.77 (0.50–1.18)	0.93 (0.68–1.29)
Health considerations	1.15 (0.87–1.51)	1.92 (1.30–2.84)**	2.12 (1.44–3.13)***	2.72 (1.80–4.10)***	1.41 (0.73–2.72)	0.56 (0.37–0.86)**	0.72 (0.52–1.01)	1.96 (1.26–3.04)**	1.05 (0.77–1.44)
Price / Affordability	0.89 (0.67–1.18)	0.80 (0.54–1.20)	0.92 (0.62–1.37)	1.11 (0.73–1.68)	1.23 (0.66–2.29)	2.93 (1.89–4.54)***	1.48 (1.06–2.09)*	3.42 (2.21–5.31)***	2.09 (1.52–2.87)***
Quality of Ingredients	0.92 (0.70–1.20)	1.50 (1.02–2.21)*	2.39 (1.62–3.51)***	2.12 (1.40–3.21)***	0.44 (0.22–0.86)*	0.48 (0.32–0.74)***	0.70 (0.50–0.97)*	0.77 (0.50–1.18)	1.21 (0.88–1.66)
Environmental considerations	0.76 (0.41–1.41)	0.74 (0.28–1.91)	1.59 (0.70–3.60)	1.17 (0.45–3.00)	0.27 (0.03–2.14)	0.44 (0.16–1.22)	0.75 (0.37–1.50)	1.27 (0.45–3.62)	1.63 (0.79–3.38)

*Odds ratios (OR) with 95% confidence intervals (CI) from multivariable logistic regression models. All models were adjusted for gender, age group, educational level, and household income. Reference categories: men (gender), ≤ 29 years (age), tertiary education (education), and > 2500 € (income). *p < 0.05, **p < 0.01, ***p < 0.001

### Discussion

This nationally representative study examined how sociodemographic factors are associated with food choice motives among Czech adults. Distinct patterns were observed across gender, education, and income groups, highlighting the multidimensional and context-dependent nature of food decision-making. Taste, price, and health considerations were the most frequently reported motives, whereas environmental aspects remained marginal.

Overall, the findings underscore the dominant role of immediate, tangible considerations in food choice, while broader sustainability-related motivations appear weakly embedded in everyday decision-making. The observed patterns are consistent with evidence from other European populations, suggesting that food choice motives are shaped by an interplay of biological, cultural, and socioeconomic factors [7, 17, 21, 22]. 

### Gender differences in food choice

Gender differences were observed for selected food choice motives. In line with the descriptive analyses, men more frequently reported taste-based motivations, whereas women placed greater emphasis on nutritional value, health considerations, price/affordability, and quality of ingredients. These patterns may reflect broader sociocultural norms surrounding food and gender identity, whereby women’s food choices are more often shaped by internalized health and body-related expectations, while men’s decisions may place greater emphasis on sensory gratification [23–25]. 

These differences should not be interpreted as biologically determined but rather as socially constructed, arising from gendered socialization processes, cultural expectations, and differences in nutrition-related knowledge and responsibilities [26]. 

### Age-related differences in food choice

In contrast to some previous studies, age-related differences in food choice motives were relatively modest in the present data [12, 19]. Descriptive analyses revealed no substantial age gradients for appearance or taste, and no clear monotonic trends for most other food choice criteria. A weak, non-significant increase in the prioritization of ingredient quality with age was observed.

Nevertheless, existing life-course models of food choice suggest that aging may be accompanied by shifts from hedonic toward health-oriented motivations, driven by physiological changes, health concerns, and changing economic circumstances [12, 19, 27]. The limited age differentiation observed in this study may therefore reflect contextual factors specific to the Czech population or cohort effects, rather than the absence of age-related processes per se.

### Socioeconomic gradients in food choice

Clear socioeconomic gradients were observed in food choice motives. Participants with lower education and income more frequently prioritized price, taste, and appearance, whereas those with higher education more often emphasized nutritional value and ingredient quality. These findings align with evidence showing that food choices among socioeconomically disadvantaged groups are shaped by affordability constraints and limited access to health-promoting food environments [28, 29]. Educational attainment may enhance nutrition knowledge, health literacy, and self-efficacy in making informed dietary decisions, while limited resources and higher relative food prices reinforce price sensitivity among lower-income groups [30–32]. 

Such disparities are embedded within commercial food systems characterized by the widespread availability and aggressive marketing of ultra-processed, price-competitive products, particularly in lower-income settings [33]. The observed patterns are consistent with findings from other European studies, highlighting the persistent role of socioeconomic inequalities in shaping food choice motives [28, 29]. 

### Environmental considerations in food choice

Environmental considerations were rarely reported and showed no strong sociodemographic patterning. This finding suggests that, within the Czech adult population, environmental impact currently plays a marginal role in everyday food decision-making. Importantly, this should not be interpreted as a lack of concern for sustainability as a broader concept, but rather as an indication that environmental considerations are weakly integrated into individual food choice motivations [14, 18]. 

Similar patterns have been documented in other Central and Eastern European contexts, where environmental aspects of food consumption tend to be less salient than in Western European populations [13, 21]. The low prominence of environmental motives may reflect limited public awareness, perceived economic trade-offs, or the absence of clear informational cues linking food products to environmental outcomes. It should also be noted that environmental considerations were assessed using a single, general item, which does not capture the full multidimensional nature of sustainability.

### Public health and policy implications

The observed socioeconomic gradients in food choice motives substantially affect public health and nutrition policy in Central and Eastern Europe. The predominance of price- and taste-driven choices among lower-educated and lower-income groups reflects persistent inequalities in food environments rooted in the socio-economic transitions of the post-socialist era. Following market liberalization in the 1990s, rapid penetration of ultra-processed and price-competitive foods, combined with widening social disparities, has contributed to the region’s escalating burden of obesity, diabetes, and other non-communicable diseases [5, 34]. Similar structural relationships between socioeconomic conditions, food environments, and dietary behaviour have been documented in Central and Eastern European contexts [35]. The findings highlight a misalignment between current sustainability-oriented policy ambitions and the motivational realities of individual food choice. Given the marginal role of environmental considerations observed in this study, policy strategies aimed at promoting sustainable diets should not assume high intrinsic consumer motivation, particularly in socioeconomically disadvantaged groups [15, 16]. Instead, effective interventions are likely to require structural approaches that integrate health, affordability, and environmental objectives, rather than relying on individual value-based behaviour change alone [36–38]. From a policy perspective, the low salience of environmental considerations underscores the challenge of translating sustainability discourse into everyday dietary decision-making. Rather than relying on individual motivation alone, integrating environmental objectives into existing health- and equity-oriented food policies may offer a more feasible pathway toward aligning public health and sustainability goals without exacerbating social inequalities.

## Limitations

This study has several limitations that should be acknowledged. First, the cross-sectional design precludes causal inference; therefore, the observed associations between sociodemographic characteristics and food choice criteria cannot be interpreted as directional or causal. Second, all data were self-reported and may be subject to recall and social desirability bias, particularly for variables related to income and health considerations. In addition, household income was not adjusted for household size, which may have introduced some misclassification of economic position, especially in larger households. Third, food choice motives were measured using single-item indicators, and environmental considerations were captured in a broad manner. This limits the ability to assess the multidimensional nature of sustainability-related motivations. Fourth, respondents were restricted to selecting up to three food choice criteria, which may have constrained the expression of more nuanced or overlapping motivations.

Although quota sampling ensured alignment with population distributions for key sociodemographic characteristics, residual selection bias cannot be entirely excluded. In this context, potential selection bias related to recruitment procedures may represent a more relevant limitation than issues of reverse causation inherent to the cross-sectional design. Furthermore, the analyses did not incorporate post-stratification weights or survey-adjusted variance estimation; as a result, standard errors may be underestimated and population-level inference should be made with caution. The findings should therefore be interpreted as associative rather than strictly population-representative estimates. In addition, the use of aggregated geographic macro-areas may have obscured finer regional heterogeneity and may introduce ecological bias when interpreting associations at the individual level. Finally, while the study provides nationally relevant data for Czechia, the findings may not be directly generalizable to populations with different cultural or socioeconomic contexts. Despite these limitations, the large sample size, standardized data collection procedures, and rigorous analytic approach strengthen the overall robustness and relevance of the findings.

## Conclusions

This nationally representative study from Czechia shows that food choice motives vary across key sociodemographic characteristics, particularly gender, education, and income. Individuals with lower education and income tended to prioritize price, taste, and appearance, whereas those with higher education were more likely to emphasize nutritional value and ingredient quality. Environmental considerations played a marginal role in food choice across all sociodemographic groups, suggesting that environmentally motivated food choices remain weakly embedded in everyday decision-making in Czechia.

These findings underscore the importance of distinguishing between sustainability as a policy goal and the specific motivations that guide individual behaviour. From a policy perspective, addressing structural and socioeconomic determinants of food choice while cautiously integrating environmental considerations may help align public health and sustainability objectives without exacerbating existing inequalities in Central and Eastern Europe.

## Supplementary Information

Supplementary Material 1.

Supplementary Material 2.

## Data Availability

The data analyzed in this study were collected as part of a sociological survey conducted by the INRES agency in 2023. Due to privacy and ethical restrictions, the dataset is not publicly available. Data may be obtained from the corresponding author upon reasonable request.
