# Supplementary material for: What drives food choices? Sociodemographic predictors in a representative Czech population sample
Source: BMC Public Health. 2026 Mar 9;26:1227. doi: 10.1186/s12889-026-26926-z (PMC13081271; doi:10.1186/s12889-026-26926-z)
Supplement: Supplementary file 1 — Supplementary Material 1. [file 12889_2026_26926_MOESM1_ESM.docx]

**Supplementary Material 1: Structured Questionnaire (English Version) and Pilot Testing of the Questionnaire**

**Pilot testing of the questionnaire**

The questionnaire was pilot-tested in a sample of 206 respondents representing different age and educational groups. The pilot study aimed to assess clarity, comprehensibility, wording, response options, and overall acceptability of the instrument.

Participants completed the questionnaire under field conditions comparable to those used in the main survey. Structured feedback was collected from respondents and interviewers regarding potential ambiguities, item interpretation, and technical aspects of administration.

Based on the pilot results, minor wording adjustments were implemented to improve clarity and reduce potential misinterpretation. No structural modifications, item deletions, or substantial changes to the questionnaire content were required. The final version of the questionnaire used in the main survey reflected these minor refinements.

**Structured Questionnaire (English Version)**

*(Instrument used in the survey, adapted to include only items used in the present analysis)*

**A1. Gender**
☐ Male
☐ Female
☐ Other / Prefer not to say

**A2. Age**
______ years

**A3. Highest completed education**
☐ Primary
☐ Secondary without graduation
☐ Secondary with graduation
☐ Tertiary

**A4. Household net monthly income**
☐ Less than 750 €
☐ 750–1500 €
☐ 1500–2500 €
☐ More than 2500 €
☐ Prefer not to say

**A5. Region of residence**
☐ Prague
☐ Central Bohemia
☐ South Bohemia
☐ Plzeň
☐ Karlovy Vary
☐ Ústí nad Labem
☐ Liberec
☐ Hradec Králové
☐ Pardubice
☐ Vysočina
☐ South Moravia
☐ Olomouc
☐ Zlín
☐ Moravian-Silesian

**A6. Size of municipality**
☐ Less than 1,000 inhabitants
☐ 1,000–10,000 inhabitants
☐ 10,000–100,000 inhabitants
☐ More than 100,000 inhabitants

**A7. Household composition**
☐ Single-person household
☐ Household with multiple adults
☐ Household with children

**B1. How often do you personally purchase food?**
(open response; interviewer coded)
Examples: daily / several times per week / weekly / less often

**B2. Do you usually read nutrition labels on food products when shopping?**
☐ Yes
☐ No

**C1. According to what criteria do you choose your food?**
*Please select up to three factors.*

☐ Appearance
☐ Taste
☐ Nutritional value
☐ Health considerations
☐ Price / Affordability
☐ Quality of ingredients
☐ Environmental considerations
☐ Other (please specify): ___________________
