# Supplementary material for: What drives food choices? Sociodemographic predictors in a representative Czech population sample
Source: BMC Public Health. 2026 Mar 9;26:1227. doi: 10.1186/s12889-026-26926-z (PMC13081271; doi:10.1186/s12889-026-26926-z)

**Supplementary Material 2 for:** What drives food choices? Sociodemographic predictors
in a representative Czech population sample.

**Table S1. Food choice criteria by gender**

Proportions (%) and p-values from chi-square tests.
Multiple responses were possible.

| Food choice criterion | Men | Women | p-value |
| --- | --- | --- | --- |
|  | n (%) | n (%) |  |
| Appearance | 191 (21.7) | 187 (20.1) | 0.390 |
| Taste | 586 (66.6) | 562 (60.3) | 0.005 |
| Nutritional value | 169 (19.2) | 251 (26.9) | <0.001 |
| Health considerations | 223 (25.3) | 345 (37.0) | <0.001 |
| Price / Affordability | 309 (35.1) | 369 (39.6) | 0.049 |
| Quality of ingredients | 240 (27.3) | 312 (33.5) | 0.004 |
| Environmental considerations | 21 (2.4) | 24 (2.6) | 0.796 |

**Table S2. Food choice criteria by age group**

Differences across age groups were tested using the chi-square test for trend.

| Food choice criterion | ≤29 years | 30–44 years | 45–59 years | ≥60 years | p-value |
| --- | --- | --- | --- | --- | --- |
|  | n (%) | n (%) | n (%) | n (%) |  |
| Appearance | 74 (19.2) | 85 (21.5) | 106 (21.8) | 112 (20.9) | 0.586 |
| Taste | 248 (64.4) | 248 (62.8) | 298 (61.2) | 348 (64.9) | 0.916 |
| Nutritional value | 82 (21.3) | 97 (24.6) | 113 (23.2) | 127 (23.7) | 0.544 |
| Health considerations | 124 (32.2) | 127 (32.2) | 155 (31.8) | 157 (29.3) | 0.326 |
| Price / Affordability | 154 (40.0) | 142 (35.9) | 191 (39.2) | 186 (34.7) | 0.203 |
| Quality of ingredients | 104 (27.0) | 118 (29.9) | 155 (31.8) | 172 (32.1) | 0.086 |
| Environmental considerations | 6 (1.6) | 12 (3.0) | 15 (3.1) | 12 (2.2) | 0.441 |

**Table S3. Food choice criteria by education level**

Differences across education levels were tested using the chi-square test for trend.

| Food choice criterion | Primary | Upper secondary – vocational | Upper secondary – general / post-secondary | Tertiary | p-value |
| --- | --- | --- | --- | --- | --- |
|  | n (%) | n (%) | n (%) | n (%) |  |
| Appearance | 34 (28.1) | 103 (20.1) | 174 (21.9) | 67 (17.4) | 0.068 |
| Taste | 87 (71.9) | 358 (69.9) | 491 (61.8) | 212 (55.2) | <0.001 |
| Nutritional value | 20 (16.5) | 58 (11.3) | 209 (26.3) | 133 (34.6) | <0.001 |
| Health considerations | 40 (33.1) | 119 (23.2) | 257 (32.3) | 152 (39.6) | <0.001 |
| Price / Affordability | 41 (33.9) | 217 (42.4) | 306 (38.5) | 114 (29.7) | <0.001 |
| Quality of ingredients | 19 (15.7) | 97 (18.9) | 272 (34.2) | 164 (42.7) | <0.001 |
| Environmental considerations | 1 (0.8) | 6 (1.2) | 23 (2.9) | 15 (3.9) | 0.022 |

**Table S4. Food choice criteria by household income**

Differences across income categories were tested using the chi-square test for trend.

| Food choice criterion | <1200 € % | 1200–2500 € % | > 2500 € % | p-value |
| --- | --- | --- | --- | --- |
|  | n (%) | n (%) | n (%) |  |
| Appearance | 77 (18.3) | 197 (21.2) | 104 (22.6) | 0.126 |
| Taste | 241 (57.4) | 589 (63.3) | 318 (69.0) | 0.002 |
| Nutritional value | 67 (16.0) | 207 (22.2) | 146 (31.7) | <0.001 |
| Health considerations | 149 (35.5) | 266 (28.6) | 153 (33.2) | 0.025 |
| Price / Affordability | 204 (48.6) | 343 (36.8) | 131 (28.4) | <0.001 |
| Quality of ingredients | 87 (20.7) | 298 (32.0) | 167 (36.2) | <0.001 |
| Environmental considerations | 7 (1.7) | 26 (2.8) | 12 (2.6) | 0.430 |

**Figure S1. Forest plots of multivariable logistic regression models for food choice criteria**
Adjusted odds ratios (OR) with 95% confidence intervals (CI) for sociodemographic determinants of individual food choice criteria:
(A) Appearance, (B) Taste, (C) Nutritional value, (D) Health considerations, (E) Price/Affordability, (F) Quality of ingredients, and (G) Environmental considerations.
Models were adjusted for gender, age group, education, and household income.
Reference categories: men (gender), ≤29 years (age), tertiary education (education), and >2500 € (income).
Odds ratios (OR) with 95% confidence intervals (CI) are plotted on a logarithmic scale.

To ensure comparability, the x-axis range (0.1–10) was kept constant across all subplots.
For *environmental considerations*, confidence intervals extend beyond the plotting area due to the small sample size and wide uncertainty.

**S1A. Appearance**


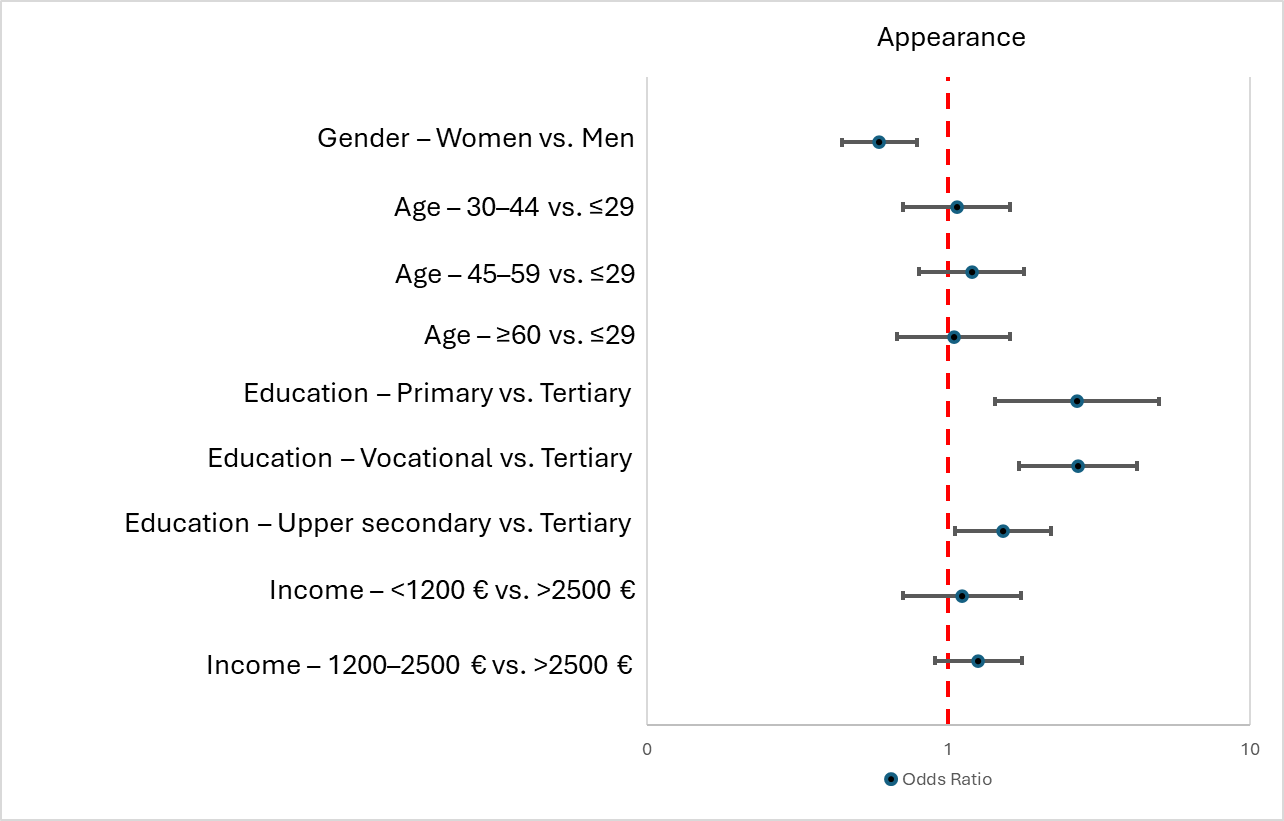


**S1B. Taste**


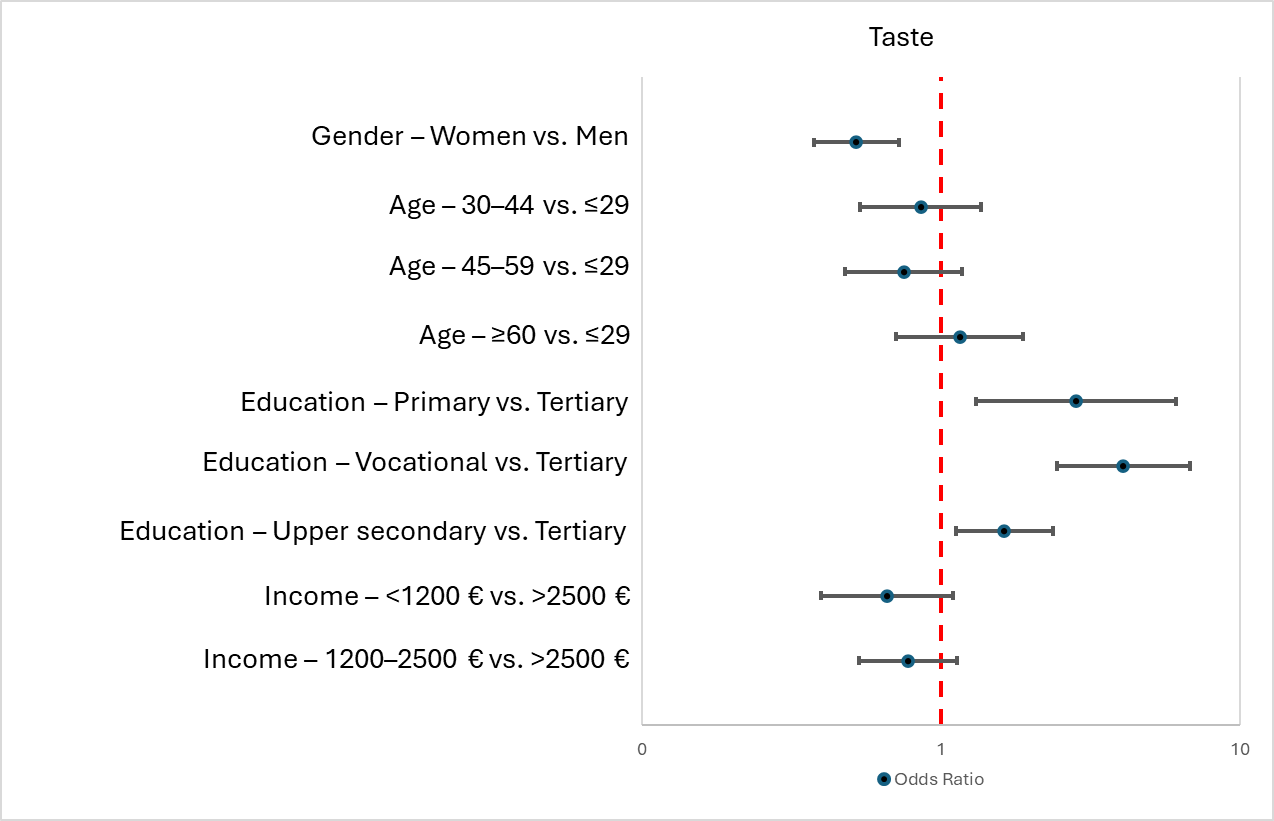


**S1C. Nutritional value**


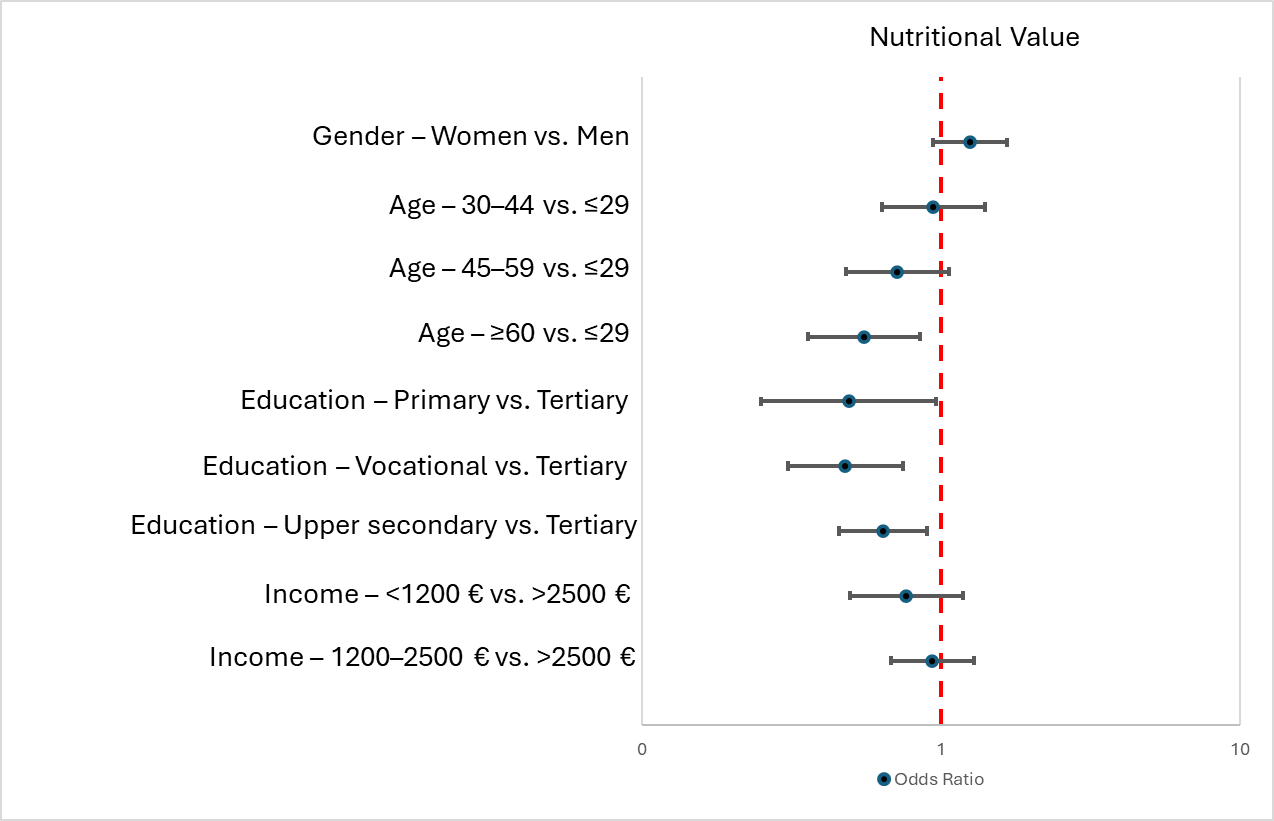


**S1D. Health considerations**


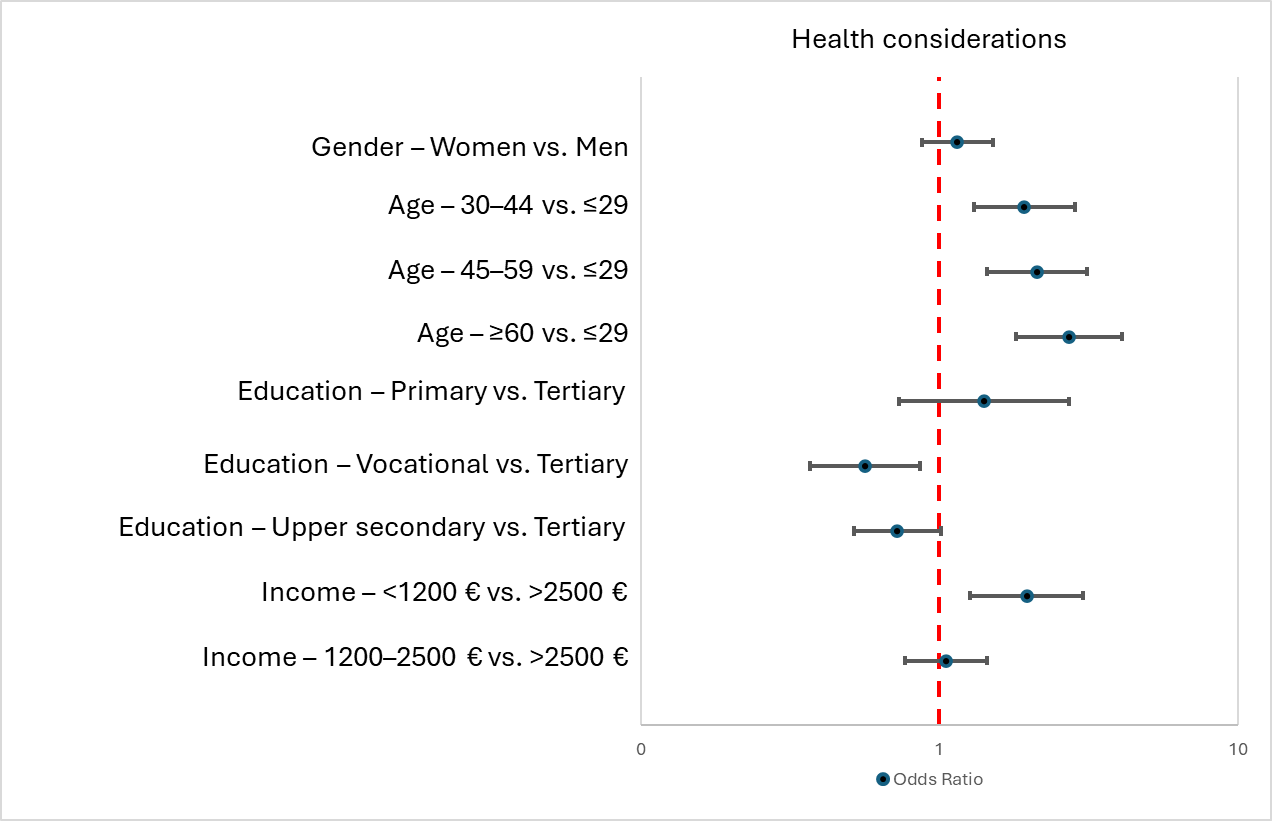


**S1E. Price / Affordability**


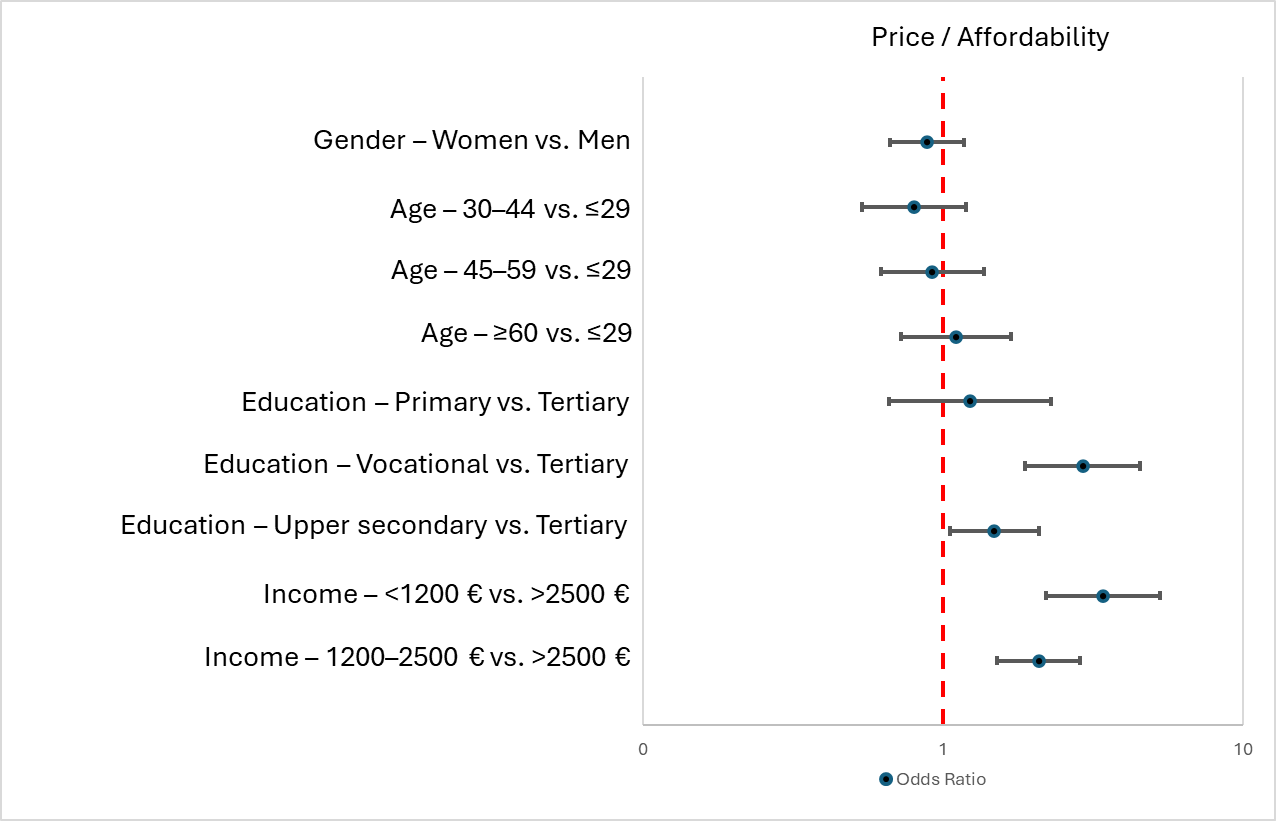


**S1F. Quality of ingredients**


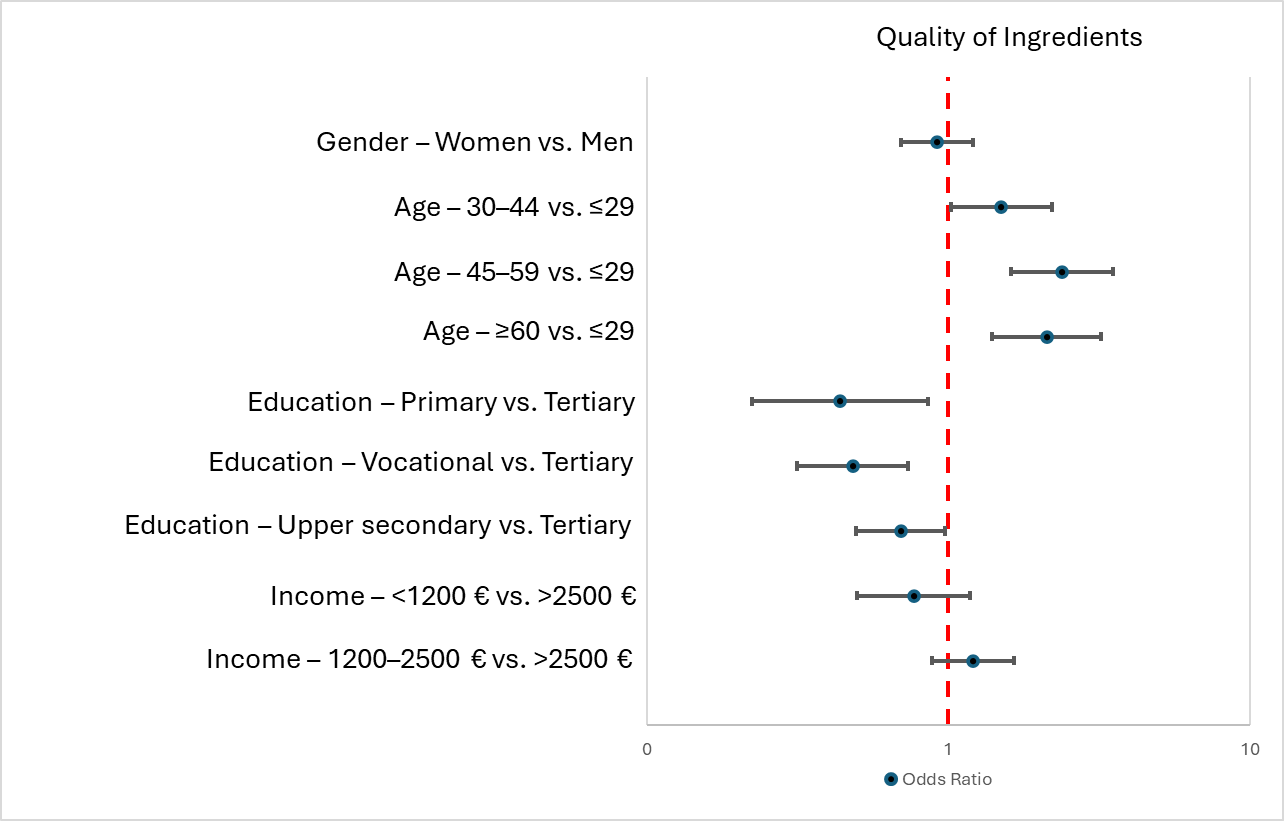


**S1G. Environmental considerations**


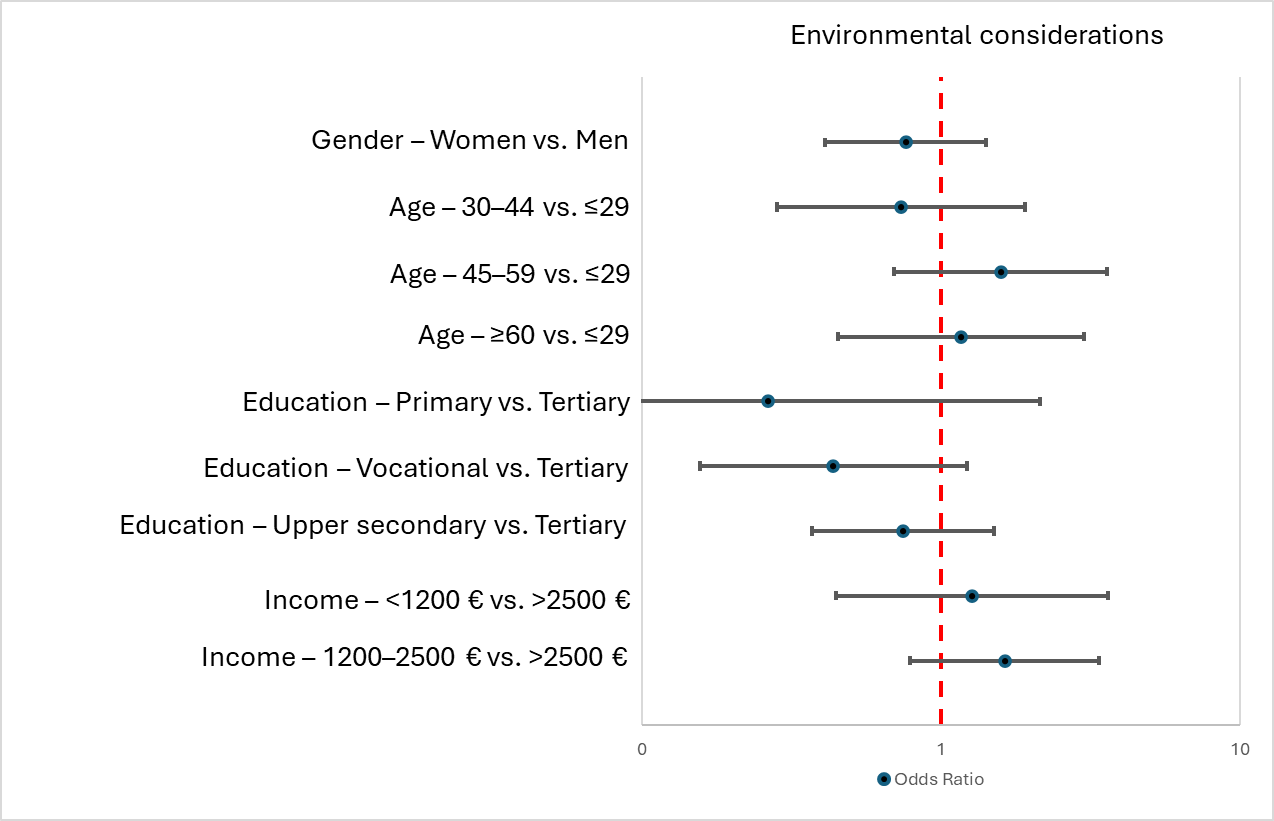

Supplement: Supplementary file 2 — Supplementary Material 2. [file 12889_2026_26926_MOESM2_ESM.docx]
